# Amyloid-β Induces Cdh1-Mediated Rock2 Stabilization Causing Neurodegeneration

**DOI:** 10.3389/fphar.2022.884470

**Published:** 2022-04-14

**Authors:** Rebeca Lapresa, Jesus Agulla, Sonia Gonzalez-Guerrero, Juan P. Bolaños, Angeles Almeida

**Affiliations:** ^1^ Institute of Functional Biology and Genomics, CSIC, University of Salamanca, Salamanca, Spain; ^2^ Institute of Biomedical Research of Salamanca, University Hospital of Salamanca, CSIC, University of Salamanca, Salamanca, Spain

**Keywords:** amyloid-β, CDK5, CDH1, ROCK2, neurodegenaration, Alzheimer’s disease

## Abstract

Alzheimer’s disease (AD) is a neurodegenerative disorder characterized by progressive cognitive decline, which is causally related to the accumulation of abnormally folded amyloid-β (Aβ) peptide and hyperphosphorylated tau protein aggregates. The dendritic spine regulator Rho protein kinase 2 (Rock2) accumulates in the brain at the earliest stages of AD and remains increased during disease progression. However, the molecular mechanism that upregulates Rock2 in AD, and its role in the disease progression, are unknown. Here, we found that oligomers of the amyloidogenic fragment 25–35 of the Aβ peptide (Aβ25-35) trigger Rock2 accumulation and activation in mouse cortical neurons in primary culture and in mouse hippocampus *in vivo*. Neuronal apoptotic death and memory impairment caused by Aβ25-35 administration were rescued by genetic and pharmacological inhibition of Rock2 activity. Mechanistically, Aβ25-35 elicited cyclin dependent kinase-5 (Cdk5)-mediated phosphorylation of Cdh1, a cofactor that is essential for the activity of the E3 ubiquitin ligase anaphase-promoting complex/cyclosome (APC/C) in neurons. Notably, phosphorylated Cdh1 was disassembled from the APC/C complex, causing its inactivation and subsequent Rock2 protein stabilization and activation. Moreover, Aβ25-35-induced neuronal apoptosis was prevented by expressing a phosphodefective form of Cdh1, but not by a phosphomimetic Cdh1. Finally, Cdh1 inactivation, using both genetic and pharmacological approaches, enhanced Aβ25-35-mediated neuronal death through a mechanism that was prevented by inhibition of Rock2 activity. These results indicate that the Cdk5-Cdh1 signaling pathway accounts for the increased Rock2 activity by amyloidogenic Aβ peptides and that this mechanism may contribute to neurodegeneration and memory loss in AD.

## Introduction

Alzheimer’s disease (AD) is the leading cause of dementia (Alzheimer’s Association, 2021), affecting around 35 million individuals worldwide hence representing an important strain on health resources (Cummings et al., 2021). However, effective disease-modifying pharmacologic therapies for AD are not currently available (Tatulian 2022). AD is causally related to the accumulation of abnormally folded amyloid-β (Aβ) peptide and hyperphosphorylated tau (pTau) protein aggregates, leading to synapse loss, neurodegeneration, and progressive cognitive decline. The widely accepted amyloid cascade hypothesis posits that the accumulation of Aβ peptides in the brain parenchyma is a key event of AD pathogenesis. Furthermore, a large body of evidence now indicates that soluble Aβ oligomers, rather than their insoluble fibrillar aggregates, are responsible for the synapto- and neurotoxicity of the Aβ peptide (Walsh et al., 2002; Karran et al., 2011). The soluble Aβ oligomers initiate a cascade of pathological pathways that trigger dendritic and synaptic alterations, which ultimately lead to synaptic plasticity impairment, neurodegeneration, and AD dementia (Selkoe and Hardy 2016; Ricciarelli and Fedele 2017). However, the underlying molecular mechanisms are not fully understood hence preventing the development of effective treatments.

The Rho protein kinase (Rock) is a serine/threonine protein kinase whose activity is regulated by the small GTPase RhoA (Strassheim et al., 2019). Once active, Rock phosphorylates various cellular substrates involved in actin cytoskeleton dynamics, hence regulating cell adhesion, contraction, migration, and division, as well as survival (Strassheim et al., 2019). The predominant brain Rock isoform is Rock2, which phosphorylates cofilin to regulate actin cytoskeleton (Zhang et al., 2006; Newell-Litwa et al., 2015), dendritic spine morphology and synaptic plasticity (Hensel et al., 2015). We recently described that Rock2 accumulation and activation in neurons triggers dendrite disruption and synapse loss, leading to memory impairment (Bobo-Jimenez et al., 2017). Moreover, Rock2 expression resulted in lower dendritic spine density (Henderson et al., 2019), whereas Rock inhibition increases the number of thin spines and filopodia, which would enhance synapse formation and neuronal plasticity (Swanger et al., 2015; Cai et al., 2021). Interestingly, dendritic dystrophy (Cochran et al., 2014; Shi et al., 2020) and synapse loss (Terry et al., 1991; Scheff et al., 2007) have both been detected in early-stage AD brains and are correlated with cognitive decline. Moreover, Rock2 protein accumulates in the neurons of early-stage human AD brain and remain elevated throughout the disease progression (Herskowitz et al., 2013). In experimental models, enhanced Rock2 activity is associated with typical AD hallmarks, such as Aβ aggregation, tau hyperphosphorylation, neuroinflammation, synaptic damage and neuronal death (Gao et al., 2019; Cai et al., 2021). However, the molecular mechanism that leads to Rock accumulation and activation in AD is unknown.

Previous results from our group have shown that the activator of the E3 ubiquitin ligase anaphase-promoting complex/cyclosome (APC/C), Cdh1, is essential for dendritic network integrity, synaptic plasticity, and neuronal survival (Almeida et al., 2005; Maestre et al., 2008; Almeida 2012; Bobo-Jimenez et al., 2017). Recently, we found that APC/C-Cdh1 targets Rock2 for proteasomal degradation in neurons, and that the loss of Cdh1 causes Rock2 stabilization and activation, triggering dendrite disruption, and dendritic spine and synapse loss in the adult brain following neurodegeneration and cognitive impairment (Bobo-Jimenez et al., 2017). Here, we hypothesized whether the regulation of Rock2 by APC/C-Cdh1 is an important mechanistic event in AD. In summary, using *in vitro* and *in vivo* experimental mouse models, we found that Aβ25-35 oligomers induce cyclin dependent kinase-5 (Cdk5)-mediated Cdh1 phosphorylation leading to APC/C inactivation and neuronal apoptosis. Interestingly, through this mechanism, Aβ25-35 triggers Rock2 accumulation and activation in neurons, inducing memory loss that could be rescued by selectively inhibiting Rock2 activity. Thus, here we identify a novel Cdh1-Rock2 pathway that is involved in Aβ neurotoxicity, which may open a new avenue for the development of therapeutic strategies to combat cognitive impairment in AD.

## Material and Methods

### Culture of Primary Cortical Neurons

Neuronal cultures were prepared from C57BL/6J mouse embryo (E14.5) cortices. Animals were maintained in specific-pathogen free facilities at the University of Salamanca, in accordance with Spanish legislation (RD53/2013) under license from the Spanish government and the European Union (2010/63/EU). Protocols were reviewed and approved by the Bioethics Committee of the University of Salamanca. All efforts were made to minimize the numbers of animals used. Neurons were seeded at 1.8 × 10^5^ cells/cm^2^ in Neurobasal medium (Invitrogen) supplemented with 2% B27 (Invitrogen) and 2 mM glutamine (Invitrogen) and incubated at 37°C in a humidified 5% CO_2_-containing atmosphere. Culture medium was replaced with fresh medium every 3 days. Neurons were used for the experiments on day 9–10 *in vitro* (Delgado-Esteban et al., 2013).

### Cell Transfections and Treatments

To achieve the silencing of proteins, we used the following commercial pre-designed small interference RNA (siRNA) (Ambion): s201147 for Cdk5; s80428 for Cdh1; s73020 for Rock2. The Silencer Select Negative Control No. 1 siRNA was used as control. Neurons were transfected with siRNA (9 nM) using Lipofectamine RNAiMAX (Invitrogen), following the manufacturer’s instructions, and used after 48 h. The efficacy of siRNAs in targeting Cdk5, Cdh1 and Rock2 is shown in Supplementary Figures S1A–C.

The active truncated amyloid-β peptide Aβ25-35 (BioNova Cientifica S.L., Madrid, Spain), was dissolved in distilled water at a concentration of 1 mg/ml and then incubated at 37°C for 3 days to induce its oligomerization (Lapresa et al., 2019). Neurons were incubated in culture medium in the absence (control) or presence of oligomerized Aβ25-35 (10 μM; BioNova Cientifica S.L.), during the time periods indicated in the Figures. When indicated, neurons were incubated in the presence of the Cdk5 inhibitor, roscovitine (10 µM Rosc; Sigma), the APC/C inhibitor, ProTAME (10 μM; Sigma), the Rock inhibitor fasudil (10 μM; Selleck Chemicals) or the Rock2 inhibitor SR3677 (10 μM; Tocris Bioscience), for the time periods indicated in the Figures.

### Western Blot Analysis and Immunoprecipitation Assay

Cells were lysed in RIPA buffer (2% sodium dodecylsulphate, 2 mM EDTA, 2 mM EGTA and 50 mM Tris pH 7.5) supplemented with phosphatase inhibitors (1 mM Na_3_VO_4_ and 50 mM NaF) and protease inhibitors (100 µM phenylmethylsulfonyl fluoride, 50 μg/ml anti-papain, 50 μg/ml pepstatin, 50 μg/ml amastatin, 50 μg/ml leupeptin, 50 μg/ml bestatin and 50 μg/ml soybean trypsin inhibitor), stored on ice for 30 min and boiled for 5 min. For *in vivo* studies, animals were sacrificed by anesthesia overdose, 5 days after intracerebroventricular (icv) injections. The brain was quickly removed from the skull, and the hippocampus was extracted and frozen in liquid N_2_. Brain tissue was homogenized in RIPA buffer, supplemented with the protease and phosphatase inhibitors, and boiled for 5 min. Extracts were centrifuged at 17,500 × *g* at 4°C for 30 min. The supernatants were collected and stored at −80°C until further use. Protein concentrations were determined with the BCA method (BCA Protein Assay kit, Thermo Fisher Scientific). Neuronal extracts were subjected to SDS-polyacrylamide gel (MiniProtean; Bio-Rad). The antibodies used were anti-Cdh1 (1:1,000; Ab-1 DH01 clone, Thermo Fisher Scientific), anti-phosphoserine (pSer; ab9332, Abcam); anti-APC3 (35/CDC27; 610455, BD Pharmingen); anti-Rock2 (1:500; D-11, Santa Cruz Biotechnology); anti-MBS (1:500, BioLegend), anti-phospho(Thr853)-MBS (1:500, MyBioSource), and anti-GAPDH (1:40000; Ambion) overnight at 4°C. GAPDH was used as loading control. After incubation with horseradish peroxidase-conjugated goat anti-rabbit IgG (Pierce, Thermo Scientific) or goat anti-mouse IgG (Bio-Rad), membranes were incubated with the enhanced chemiluminescence SuperSignal West Dura (Pierce) for 5 min or Immobilon Western Chemiluminiscent HRP Substrate (Merck Millipore; Darmstadt, Germany) for 1 min, before exposure to Kodak XAR-5 film for 1–5 min, and the autoradiograms were scanned (Veas-Perez de Tudela et al., 2015b).

For immunoprecipitation of endogenous Cdh1, neurons were lysed in RIPA buffer supplemented with the phosphatase and protease inhibitor cocktail indicated above. Cell extracts were clarified by centrifugation, and the supernatants (100 μg protein) were incubated with anti-Cdh1 (1 μg) for 4 h at 4°C, followed by the addition of 20 μL of protein A-agarose (GE Healthcare Life Sciences) for 2 h at 4°C. Inmunoprecipitates were washed with lysis buffer and proteins detected by western blot analysis (Maestre et al., 2008).

### Cdk5 Activity Assay

Neurons were lysed in ice-cold buffer containing 50 mM Tris (pH 7.5), 150 mM NaCl, 2 mM EDTA, 1% NP-40, supplemented with the phosphatase and protease inhibitors cited above. After clearing debris by centrifugation, extracts (200 µg protein) were incubated with anti-Cdk5 (1 µg) for 4 h, at 4°C, followed by the addition of 30 µL of protein A-sepharose (GE Healthcare Life Sciences) for 2 h, at 4°C. Immunoprecipitates were washed in lysis buffer and resuspended in kinase buffer (50 mM Hepes pH 7.5, 10 mM MgCl_2_, 1 mM EDTA and 0.1 mM dithiothreitol) containing 20 µM ATP, 2 µCi of [γ-^32^P]ATP and histone H1 (1 mg/ml; Sigma). Samples were subjected to SDS-polyacrylamide gel (12%) electrophoresis and transferred proteins were visualized by autoradiography or blotted with anti-Cdk5 (Lapresa et al., 2019).

### APC/C Ubiquitin Ligase Activity Assay

Active APC/C was immunoprecipitated from neurons using monoclonal anti-APC3 antibody (BD Pharmingen) and immobilized on Dynabeads Protein A (Invitrogen). Immunoprecipitates were incubated at 37°C in 10 µL of buffer (0.1 M KCl, 2.5 mM MgCl_2_, 2 mM ATP, 7.5 µg ubiquitin, 0.3 mM dithiothreitol, 135 mM MG132, 1 mM ubiquitin aldehyde, 2.5 mM His-UbcH10 and 2.5 µM UbcH5a in 20 mM Tris-HCl, pH 7.5) containing 2.5 µL of APC/C beads and 1 µL of [^35^S]cyclin B1. Reactions were stopped at the indicated time points with SDS sample buffer, mixtures resolved by SDS-polyacrylamide gel electrophoresis and visualized by phosphorimaging. APC/C activity was expressed as densitometry of the bands using ImageJ 1.48u4 software (National Institutes of Health, United States) (Delgado-Esteban et al., 2013).

### Neuronal Apoptosis Determination by Flow Cytometry and Active Caspase-3 Fluorimetric Detection

Neurons were carefully detached from the plates using 1 mM EDTA tetrasodium salt in PBS (pH 7.4) at room temperature and neuronal apoptosis was assessed by flow cytometry. Neurons were stained with annexin V-allophycocyanin (APC; Becton Dickinson Biosciences, New Jersey, United States) and 7-aminoactinomycin D (7-AAD; Becton Dickinson Biosciences) in binding buffer (100 mM HEPES, 140 mM NaCl, 2.5 mM CaCl2) to quantitatively determine the percentage of apoptotic neurons by flow cytometry. The annexin V-APC-stained neurons that were 7-AAD-negative were apoptotic. Triplicates obtained from four different neuronal cultures were analysed on a FACScalibur flow cytometer (15 mW argon ion laser tuned at 488 nm. CellQuest software, Becton Dickinson Biosciences) (Gomez-Sanchez et al., 2011).

A fluorimetric caspase-3 assay kit (Sigma) was used following the manufacture’s protocol. Cells were lysed with 50 mM HEPES, 5 mM CHAPS, 5 mM DTT, pH 7.4 for 20 min on ice, and the assay buffer containing the Ac-DEVD-AMC (acetyl-Asp-Glu-Val-Asp-7-amino-4-methylcoumarin) substrate (20 mM HEPES, 2 mM EDTA, 0.1% CHAPS, 5 mM DTT, 16 µM Ac-DEVD-AMC, pH 7.4) was added. Aliquots of 200 µL were transferred to a 96-wells plate and the fluorescence was recorded at 5 min intervals for 30 min at 37°C using a Fluoroskan Ascent FL (Thermo Scientific) fluorimeter (excitation: 360 nm, emission: 460 nm). Caspase-3 activity was determined as (7-amino-4-methylcoumarin) AMC release rate extrapolating the slopes to those obtained from the AMC standard curve. Results were expressed as pmol/h/µg protein (Sanchez-Moran et al., 2020).

### Immunocytochemistry

Neurons grown on glass coverslips were fixed with 4% (w/v, in PBS) paraformaldehyde for 30 min and immunostained with mouse anti-Cdh1 (1:250; Ab-1 DH01 clone, Thermo Fisher Scientific), mouse anti-Map2 (1:500; Sigma), rabbit anti-cleaved caspase-3 (1:300; Cell Signaling Technology), mouse anti-Rock2 (1:300; D-11, Santa Cruz Biotechnology), and rabbit anti-Map2 (1:500; Abcam). Immunolabeling was detected using IgG-Cy2 (1:500) or IgG-Cy3 (1:500) secondary antibodies (Jackson ImmunoResearch Inc.). Nuclei were stained with 6-diamidino-2-phenylindole (DAPI, Sigma D9542). Coverslips were washed, mounted with SlowFace light antifade reagent (Invitrogen) and examined under an Olympus IX81 Spinning disk confocal microscope (Olympus) (Lapresa et al., 2019).

### Mice and Mouse Model of Single Intracerebroventricular Injection of Aβ25-35

Mice were maintained in specific-pathogen free facilities at the University of Salamanca, in accordance with Spanish legislation (RD53/2013) under license from the Spanish government and the European Union (2010/63/EU). Mouse model protocols were approved by the Bioethics Committee of the University of Salamanca. All efforts were made to minimize the numbers of animals used. The study included C57BL/6J male mice divided in three experimental groups, either receiving saline (control), oligomerized Aβ25-35 (Aβ25-35) or oligomerized Aβ25-35 plus Rock2 inhibitor (Aβ25-35 + SR3677).

Stereotaxic injections were performed as previously done (Lapresa et al., 2019). Twelve-week-old male mice were anesthetized by inhalatory induction (4%) and maintained (2.5%) with sevoflurane (Sevorane; Abbot) in a gas mixture of 70% N_2_O, 30% O_2_, using a gas distribution column (Hersill H-3) and a vaporizer (InterMed Penlons Sigma Delta). Mice were placed in a stereotaxic alignment system (Model 1900; David Kopf Instruments) with digital read out (Wizard 550, Anilam). Injection was performed into the right ventricle at coordinates: 0.22 mm posterior to bregma, 1 mm lateral to midline, and 2.5 mm ventral to dura, using a 5-µL Hamilton syringe (Microliter 65RN, Hamilton) with a 26 S needle (type 2 tip). Either 4 μL of saline (control) or oligomerized Aβ25-35 (9 nmol) were injected using a mini-pump (UltraMicroPump III, World Precision Instruments) and a digital controller (Micro4 UMC4; World Precision Instruments), at a rate of 0.8 μL/min during 5 min. The syringe was left in place for 10 min before slowly retracting it to allow for Aβ infusion and to prevent reflux. Wounds were sutured, and animals were allowed to recover from anesthesia in cages placed on a 37°C thermostatic plate (Plactronic Digital, 25 × 60, JP Selecta). When indicated, animals were intracerebroventricularly injected with both oligomerized Aβ25-35 and Rock2 inhibitor, SR3677 (2 mg/kg, Tocris Bioscience) (Herskowitz et al., 2013). Hippocampal samples from these animals (*n* = 3 male mice per experimental group) were collected for western blot analysis at 1, 3 and 5 days after injections. In addition, functional studies were performed, as described below (*n* = 7 male mice per experimental condition).

### Behavioral Tests

Male mice (3 months old) were left to acclimate in the room for no less than 45 min at the same time of day. Tracking was carried out one at a time using a video-tracking system (ANY-maze, Stoelting Europe), and the apparatus was wiped thoroughly between each mouse session to avoid olfactory cues.

*Open-field Test (OF)*. Mice were placed in an ANY-box core (40 × 40 cm). The arena of the box was divided in two zones, namely border (8 cm wide) and centre (a 20 × 20 cm square at the centre of the arena). The animals were allowed to freely explore the field for 10 min, and the distance, time and number of entries to the centre area were recorded. Mice were tested 5 days after icv injection. (Jimenez-Blasco et al., 2020).

*Novel object recognition task (NORT)*. The test consisted of two phases. A familiarization phase, where mice were placed in the same ANY-box core with two identical objects located in the upper left and lower right corner of the arena 8 cm away frow the walls. The animals were allowed to freely explore the objects for 5 min and returned to their cages immediately afterwards for 30 min. Short-term memory was evaluated in the recognition phase, in which the animals were returned to the arena, where one of the objects was replaced for a novel one. We registered the number of explorations and the time exploring each object were recorded, and analyzed the discrimination index using the formula DI=(t_N_–t_F_)/(t_N_ + t_F_), where t_N_ is the time spent exploring the new object, and t_F_ is the time spent exploring the familiar one. Mice were tested 5 days after icv injection (Vicente-Gutierrez et al., 2019).

*Barnes Maze*. The apparatus consisted of a grey circular platform, 120 cm in diameter elevated 90 cm above the floor. Along its perimeter were 20 evenly spaced holes. The maze has one removable escape box that could be fitted under any of these holes and was filled with the animal bedding before each experiment. Black and white patterned pictures were used as spatial visual cues. All sessions were performed under a room lightning of 400 lux to increase the mouse aversion for the platform. The test consisted of three phases. First, the habituation phase, where the animals were left to explore the platform freely for 5 min 1-day before the training sessions. Afterwards, the animals underwent the training phase where they were allowed to locate the scape hole for a maximum of 5 min, for 3 days with 4 sessions per day. Finally, for the probe phase, mice were tested for spatial memory. In this session the escape box was removed, and the platform was virtually divided into four quadrants, each containing five holes. Mice were allowed to explore the maze for 5 min and the time spent in the quadrant that previously contained the escape box was quantified. The animals were tested before and 5 days after icv injection (Islam et al., 2021).

### Statistical Analysis

Results are expressed as mean ± SEM. A one-way or two-way ANOVA with Tukey’s post hoc test was used to compare values between multiple groups, and a two-tailed, unpaired Student’s t-test was used for two-group comparisons. In all cases, *p* < 0.05 were considered significant. Statistical analyses were performed using SPSS Statistics 24.0 for Macintosh (IBM).

## Results

**FIGURE 1:**
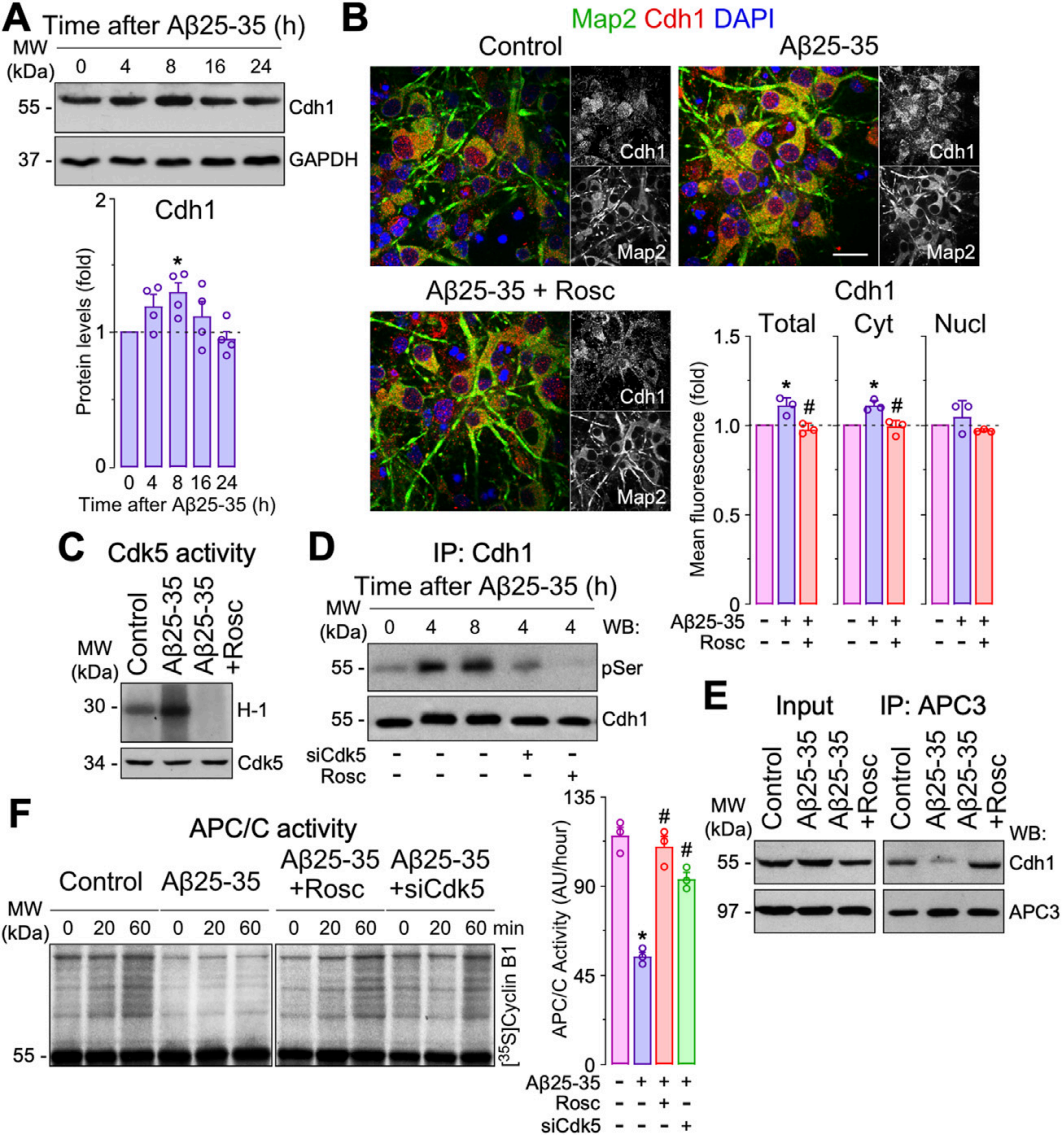
Amyloid-β (Aβ)-induced Cdh1 phosphorylation disassembles Cdh1 from APC3 leading to APC/C inactivation. Primary cortical neurons were incubated in culture medium in the absence (control) or the presence of oligomerized Aβ25-35 (10 μM). When indicated, medium was supplemented with roscovitine (10 μM; Rosc). **(A)** Cdh1 western blot analysis in neurons at different time points of Aβ25-35 incubation (GAPDH, loading control). Cdh1 western blot bands were quantified by densitometry and data were expressed as the fold change relative to 0 time (*n* = 4 neuronal cultures). **(B)** Cdh1 and Map2 (neuronal marker) immunocytochemical analysis in neurons treated with Aβ25-35 and roscovitine for 8 h. Scale bar = 20 µm. Total, nuclear and cytosolic Cdh1 mean fluorescence were quantified and data were expressed as the fold change relative to control (*n* = 4 neuronal cultures). **(C)** Western blot analysis showing Cdk5 activity in neurons at 2 h of Aβ25-35 incubation. **(D)** Neurons on day 6 *in vitro* were transfected with siRNA control (9 nM) or with siRNA against Cdk5 (siCdk5; 9 nM) for 2 days and then treated with Aβ25-35. Immunoprecipitated Cdh1 followed by pSerine (pSer) western blot analysis in neurons at different time points of Aβ25-35 incubation. Representative blots are shown. Protein abundance quantification from three different neuronal cultures is shown in Supplementary Figure S1D. **(E)** Coimmunoprecipitation assay showing that Aβ25-35 (4 h of incubation) disrupted Cdh1 and APC3 interaction in neurons, which was prevented by roscovitine. **(F)** APC/C activity in neurons at 4 h of Aβ25-35 incubation, as assessed by the ability of neuronal extracts to ubiquitylate, *in vitro*, 35S-cyclin B1. Time (min) indicates the reaction time of incubation with 35S-cyclin B1. APC/C activity was expressed as densitometry of the bands (60 min) from three different neuronal cultures (*n* = 3). Data are mean ± SEM for the indicated number of neuronal cultures. *p < 0.05 *versus* control; #*p* < 0.05 *versus* Aβ25-35.

**Aβ-induced Cdh1 phosphorylation causes APC/C inactivation and neuronal apoptosis.** To understand the potential role of the Cdh1-Rock2 pathway in Aβ neurotoxicity, we first analyzed the effect of Aβ on APC/C-Cdh1 activity. Cdh1 is the main activator of APC/C in neurons, where it is regulated by autoubiquitination, Cdk5-mediated phosphorylation and subcellular localization (Maestre et al., 2008; Almeida 2012). Treatment of neurons with oligomers of the amyloidogenic fragment Aβ25-35 time-dependently enhanced Cdh1 protein levels (Figure 1A) until 8 h of incubation, especially in the cytosol (Figure 1B); this effect was prevented by roscovitine, a cyclin dependent kinase-5 (Cdk5) inhibitor (Figure 1B), suggesting that Aβ25-35 might cause Cdh1 phosphorylation (Maestre et al., 2008; Veas-Perez de Tudela et al., 2015b; Fuchsberger et al., 2016). In accordance with our previous results (Lapresa et al., 2019), Aβ25-35 rapidly (2 h) activated Cdk5 (Figure 1C), resulting in a time-dependent phosphorylation of Cdh1 (Figure 1D and Supplementary Figure S1D). Since Cdh1 phosphorylation at Cdk5-cognate residues disrupts Cdh1 assembly from its APC/C complex core protein APC3, leading to APC/C-Cdh1 inactivation in neurons (Veas-Perez de Tudela et al., 2015b), we next assessed whether these effects could be mimicked by Aβ25-35 treatment. As shown in Figure 1E, APC3-Cdh1 interaction was disrupted by Aβ25-35, an effect that was rescued with roscovitine. Moreover, Aβ25-35 treatment caused APC/C inactivation, as revealed by the decreased ubiquitination of its well-known substrate, cyclin B1 (Zachariae et al., 1998), in a manner that could be prevented by roscovitine and Cdk5 knock down (siCdk5) (Figure 1F). Thus, Aβ25-35-induced Cdk5 activation promotes the accumulation of phosphorylated (inactive) Cdh1 and its disassembly from APC3, leading to APC/C-Cdh1 inactivation.

**FIGURE 2:**
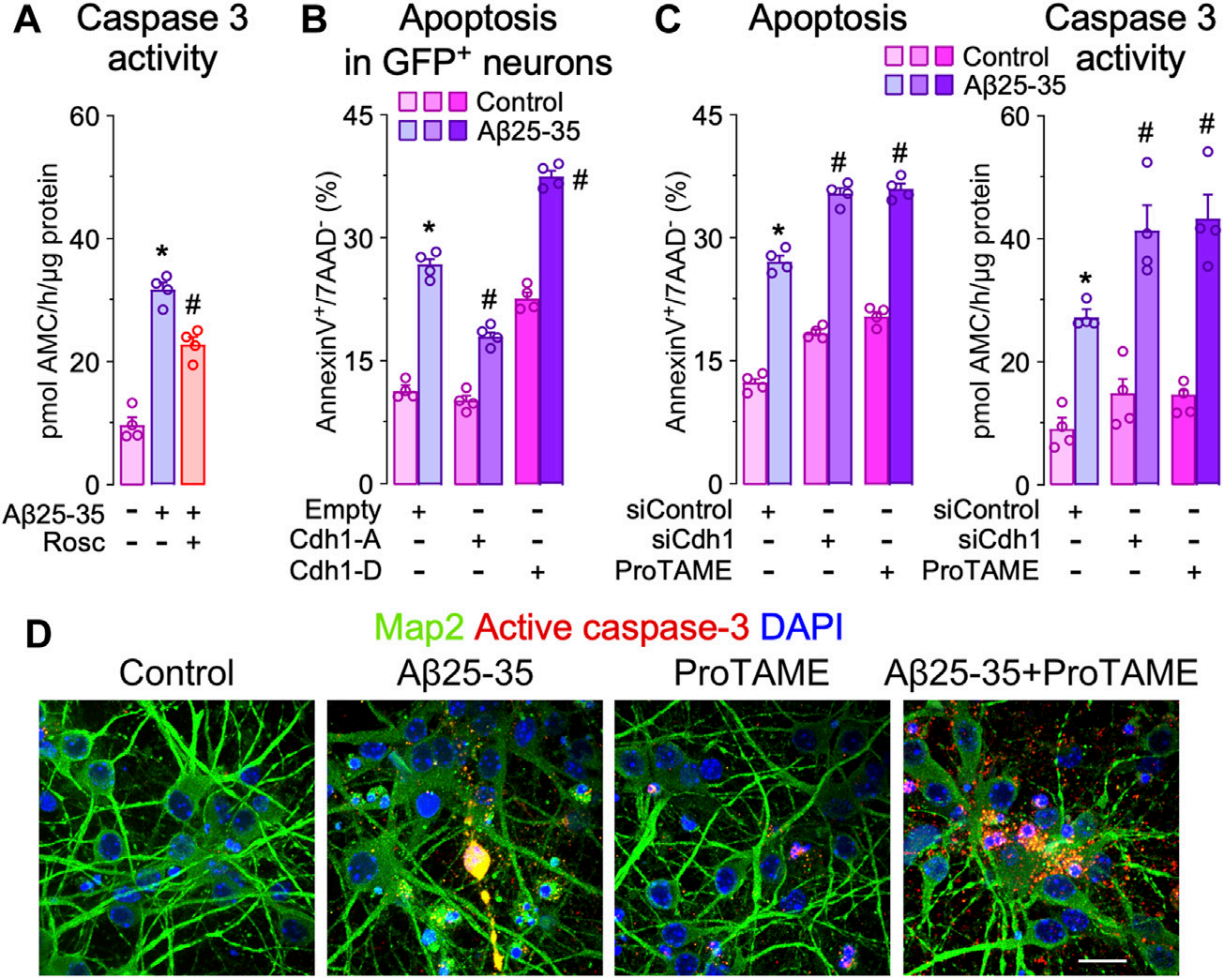
Cdh1 phosphorylation mediates Aβ25-35 neurotoxicity. Primary cortical neurons were incubated in culture medium in the absence (control) or the presence of oligomerized Aβ25-35 (10 μM). When indicated, medium was supplemented with roscovitine (10 μM; Rosc) or ProTAME (10 µM). **(A)** Caspase-3 activity determination in neurons at 24 h of incubation with Aβ25-35 and roscovitine (*n* = 4 neuronal cultures). **(B)** Neurons on day 6 *in vitro* were transfected with empty vector or vectors co-expressing GFP and either the phosphodefective (Cdh1-A) or the phosphomimetic (Cdh1-D) forms of Cdh1 and were subjected to Aß25-35 exposure for 24 h. Apoptosis was measured by flow cytometry in GFP^+^ (transfected) neurons (*n* = 4 neuronal cultures). **(C)** Neurons on day 6 *in vitro* were transfected with siRNA control (9 nM) or with siRNA against Cdh1 (siCdh1; 9 nM) for 2 days and then treated with Aβ25-35 oligomerized and ProTAME. Neuronal apoptosis and caspase-3 activity were analyzed in neurons at 24 h of incubation with Aβ25-35 (*n* = 4 neuronal cultures). **(D)** Active caspase-3 and Map2 (neuronal marker) immunocytochemical analysis in neurons treated with Aβ25-35 and ProTAME for 24 h. Scale bar = 20 µm. Data are mean ± SEM for the indicated number of neuronal cultures. *p < 0.05 *versus* control; #*p* < 0.05 *versus* Aβ25-35.

Given that the inactivation of APC/C-Cdh1 caused by Cdh1 phosphorylation promotes neuronal apoptosis (Maestre et al., 2008; Veas-Perez de Tudela et al., 2015b; Veas-Perez de Tudela et al., 2015a), we next evaluated the impact of Cdh1 in Aβ25-35 toxicity. As shown in Figure 2A, Aβ25-35-induced caspase 3 activation that was partially prevented by roscovitine. Then we investigated whether Cdh1 phosphorylation by Cdk5 impacted in Aβ25-35 neurotoxicity. To this end, a phosphomimetic (Cdh1-D) or a phosphodefective (Cdh1-A) form of Cdh1 were expressed in neurons followed by Aβ25-35 treatment. As previously described, Cdh1-D resulted in neuronal apoptosis (Maestre et al., 2008; Veas-Perez de Tudela et al., 2015b). In contrast, Cdh1-A prevented Aβ25-35-induced neuronal apoptosis (Figure 2B and Supplementary Figure S2A). Moreover, inhibition of APC/C using ProTAME and Cdh1 knock down (siCdh1) enhanced Aβ25-35-induced neurotoxicity, as revealed by the increased neuronal apoptosis and caspase 3 activation (Figures 2C,D and Supplementary Figure S2A–C). Thus, Cdk5-mediated Cdh1 phosphorylation triggers APC/C inhibition, which is important in Aβ25-35 neurodegeneration.

**FIGURE 3:**
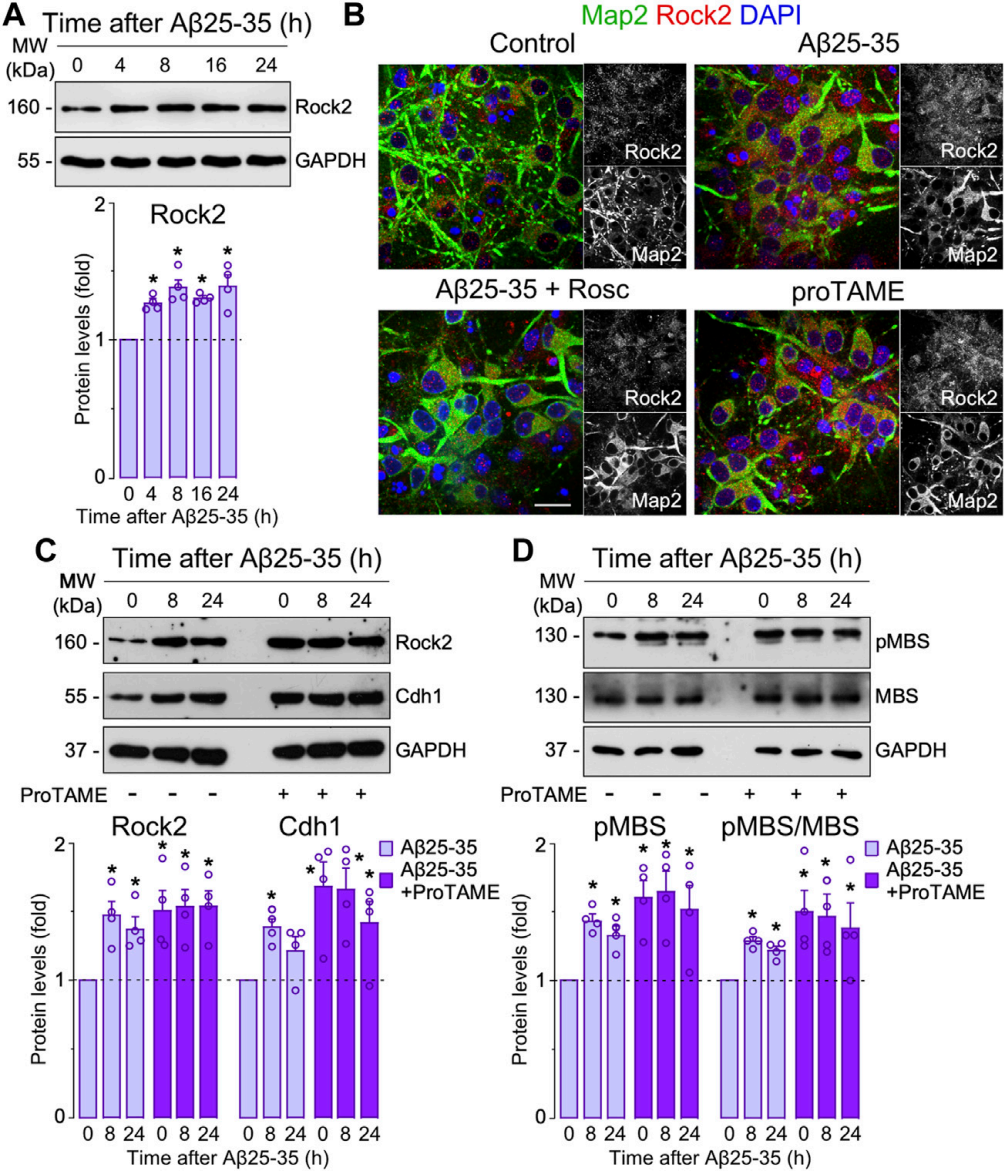
Amyloid-β (Aβ)-induced APC/C-Cdh1 inactivation triggers Rock2 stabilization and activation. Primary cortical neurons were incubated in culture medium in the absence (control) or the presence of Aβ25-35 oligomerized (10 μM). When indicated, medium was supplemented with roscovitine (10 μM; Rosc) or ProTAME (10 µM). **(A)** Rock2 western blot analysis in neurons at different time points of Aβ25-35 incubation (GAPDH, loading control). Rock2 western blot bands were quantified by densitometry and data were expressed as the fold change relative to 0 time (*n* = 4 neuronal cultures). **(B)** Rock2 and Map2 (neuronal marker) immunocytochemical analysis in neurons from one culture treated with Aβ25-35 or ProTAME for 24 h. Scale bar = 20 µm. **(C)** Rock2 and Cdh1 western blot analysis in neurons at different time points of Aβ25-35 and ProTAME incubation (GAPDH, loading control). Rock2 and Cdh1 western blot bands were quantified by densitometry and data were expressed as the fold change relative to 0 time in the absence of ProTAME (*n* = 4 neuronal cultures). **(D)** MBS and pMBS western blot analysis in neurons at different time points of Aβ25-35 and ProTAME incubation (GAPDH, loading control). MBS and pMBS western blot bands were quantified by densitometry and data were expressed as the fold change relative to 0 time in the absence of ProTAME. Data are mean ± SEM for the indicated number of neuronal cultures. *p < 0.05 *versus* 0 time of Aβ25-35 incubation (control).

**FIGURE 4:**
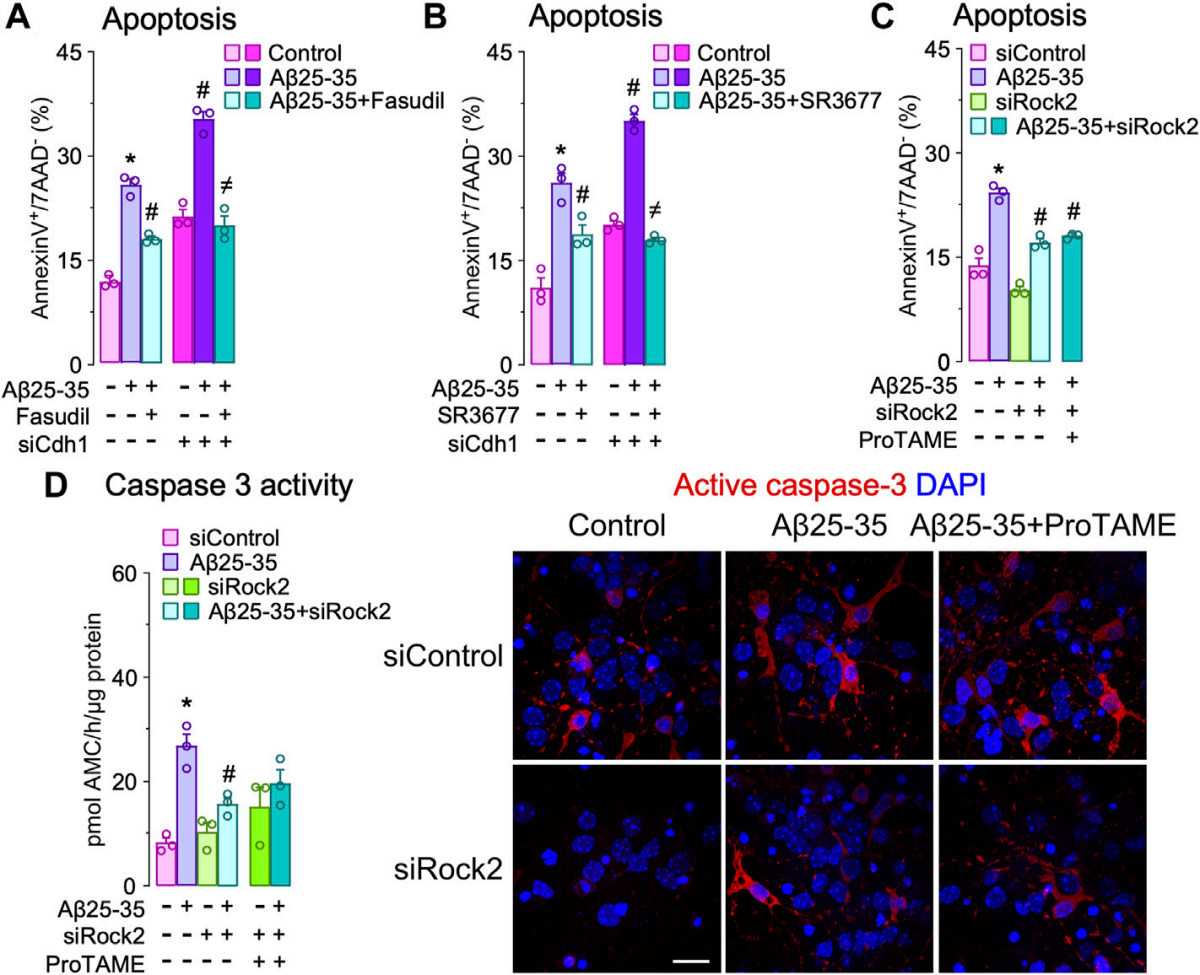
Cdh1-induced Rock2 activation is involved in amyloid-β (Aβ) neurotoxicity. Primary cortical neurons were incubated in culture medium in the absence (control) or the presence of oligomerized Aβ25-35 (10 μM). When indicated, medium was supplemented with fasudil (10 µM), Rock2 inhibitor SR3677 (10 µM) or ProTAME (10 µM). **(A,B)** Neurons on day 6 *in vitro* were transfected with siRNA control (9 nM) or with siRNA against Cdh1 (siCdh1; 9 nM) for 2 days and then treated with oligomerized Aβ25-35 and **(A)** fasudil or **(B)** SR3677. Neuronal apoptosis was analyzed in neurons at 24 h of incubation with Aβ25-35 (*n* = 3 neuronal cultures). **(C,D)** Neurons on day 6 *in vitro* were transfected with siRNA control (9 nM) or with siRNA against Rock2 (siRock2; 9 nM) for 2 days and then treated with Aβ25-35 oligomerized and ProTAME. **(C)** Neuronal apoptosis and **(D)** caspase-3 activity were analyzed in neurons at 24 h of incubation with Aβ25-35 (*n* = 3 neuronal cultures). Active caspase-3 immunocytochemical analysis in neurons treated with Aβ25-35 and ProTAME for 24 h. Data are mean ± SEM for the indicated number of neuronal cultures. *p < 0.05 *versus* control; #*p* < 0.05 *versus* Aβ25-35; ≠*p* < 0.05 *versus* Aβ25-35 + ProTAME.

**Aβ-induced APC/C- Cdh1 inactivation triggers Rock2 stabilization and activation leading to neuronal apoptosis.** Next, we sought to identify the APC/C-Cdh1 target that is involved in Aβ25-35 neurotoxicity. We specifically focused on Rock2, given that it is a well-known APC/CCdh1 substrate (Bobo-Jimenez et al., 2017) that accumulates in the brain of AD patients (Herskowitz et al., 2013). To do so, we first treated neurons with Aβ25-35 and found that Rock2 protein levels time-dependently increased (Figure 3A) through a mechanism that could be prevented with roscovitine (Figure 3B). Moreover, inactivation of APC/C-Cdh1 with ProTAME promoted the accumulation of its cognate targets, Cdh1 and Rock2, an effect that was maintained during all the time of Aβ25-35 presence (Figure 3C). To ascertain whether Aβ25-35-induced Rock2 accumulation was translated into Rock2 activity, we measured the Thr853 phosphorylation of the Rock2-subtrate myosin phosphatase myosin-binding subunit (MBS) (Tanaka et al., 2006; Bobo-Jimenez et al., 2017). As shown in Figure 3D, Aβ25-35 increased MBS phosphorylation, indicating increased Rock2 activity, and effect that was potentiated by ProTAME. These results confirm that Aβ25-35 inactivates APC/C-Cdh1 to regulate Rock2 activity (Bobo-Jimenez et al., 2017). We then aimed to investigate whether the Cdh1-Rock2 axis impacts on Aβ25-35 neurotoxicity. To do this, neurons were treated with the clinically approved Rock inhibitor, fasudil, and with the Rock2 inhibitor, SR3677. As shown in Figures 4A,B, these treatments partially rescued Aβ25-35-induced neuronal apoptosis. Furthermore, either fasudil or SR3677 reduced Aβ25-35 neurotoxicity in Cdh1-knocked down neurons (Figures 4A,B). Neuronal viability was not affected by fasudil or SR3677 treatments (Supplementary Figure S2D). Although SR3677 demonstrated an approximately eightfold selectivity of Rock2 over Rock1, at a concentration of 10 µM it is likely that SR3677 also inhibits Rock1 (Feng et al., 2008). Next, we specifically knocked down Rock2 in neurons (siRock2), which did not affect Rock1 levels (Supplementary Figure S1C). Interestingly, Rock2 knock down also protected neurons against Aβ25-35-induced apoptotic death (Figures 4C,D), an effect that was mimicked by the APC/C-Cdh1 inhibitor ProTAME (Figure 4D). Altogether, these results indicate that Aβ25-35-induced Cdh1 phosphorylation inactivates APC/C-Cdh1, which results in Rock2 stabilization and activation, eventually leading to neuronal apoptosis.

**FIGURE 5:**
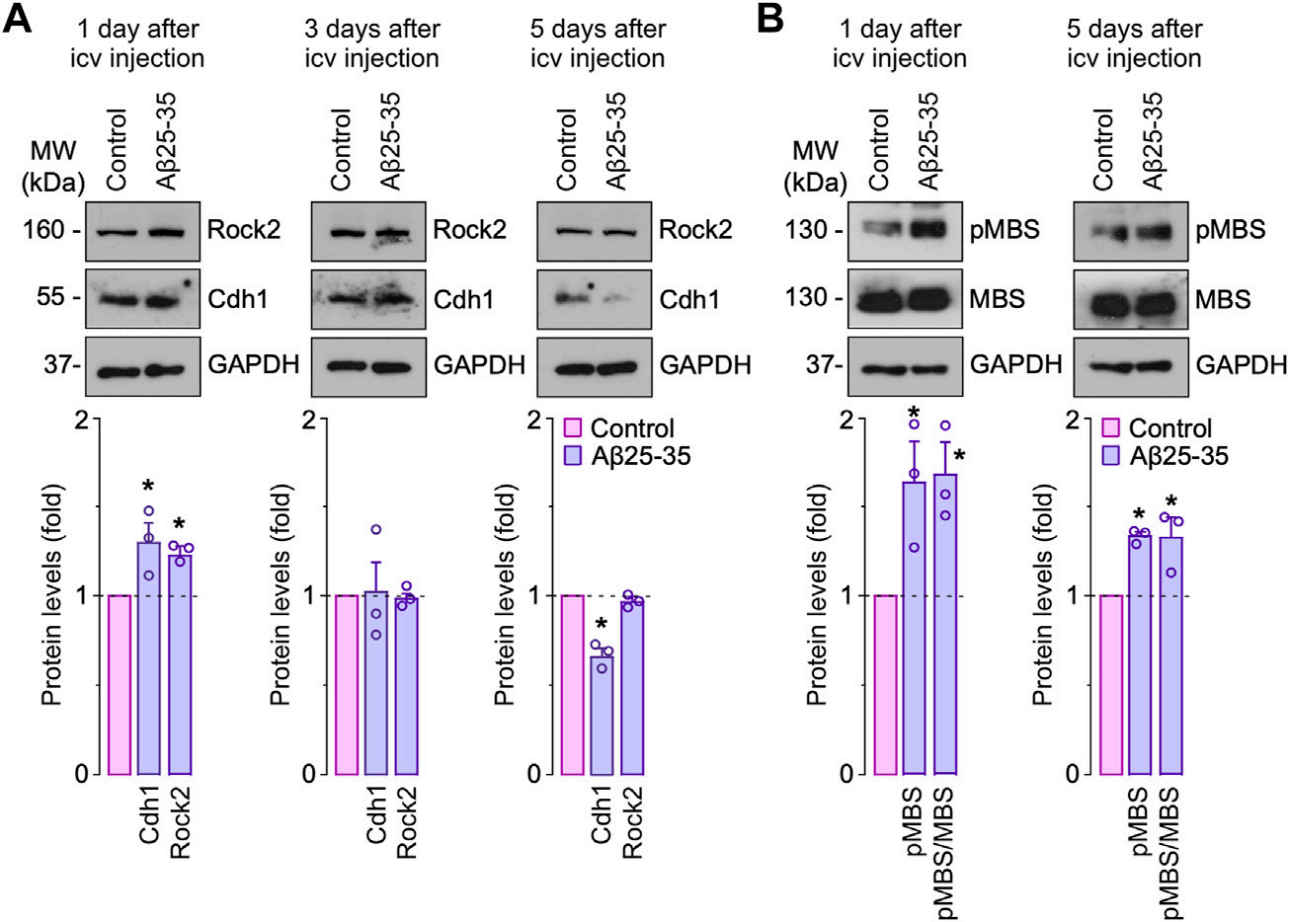
Amyloid-β (Aβ) promotes Rock2 stabilization and activation in the hippocampus *in vivo*. Intracerebroventricular stereotactic injections of saline (control) or oligomerized Aβ25-35 (9 nmol) were performed into 12-week-old male mice. Hippocampus extracts were obtained at different days after injections. **(A)** Rock2 and Cdh1 western blot analysis in hippocampus extracts (GAPDH, loading control). Rock2 and Cdh1 western blot bands were quantified by densitometry and results were expressed as the fold change relative to control. **(B)** MBS and pMBS western blot analysis in hippocampus extracts (GAPDH, loading control). MBS and pMBS western blot bands were quantified by densitometry and results were expressed as the fold change relative to control. Data are mean ± SEM from 3 mice. *p < 0.05 *versus* control.

**FIGURE 6:**
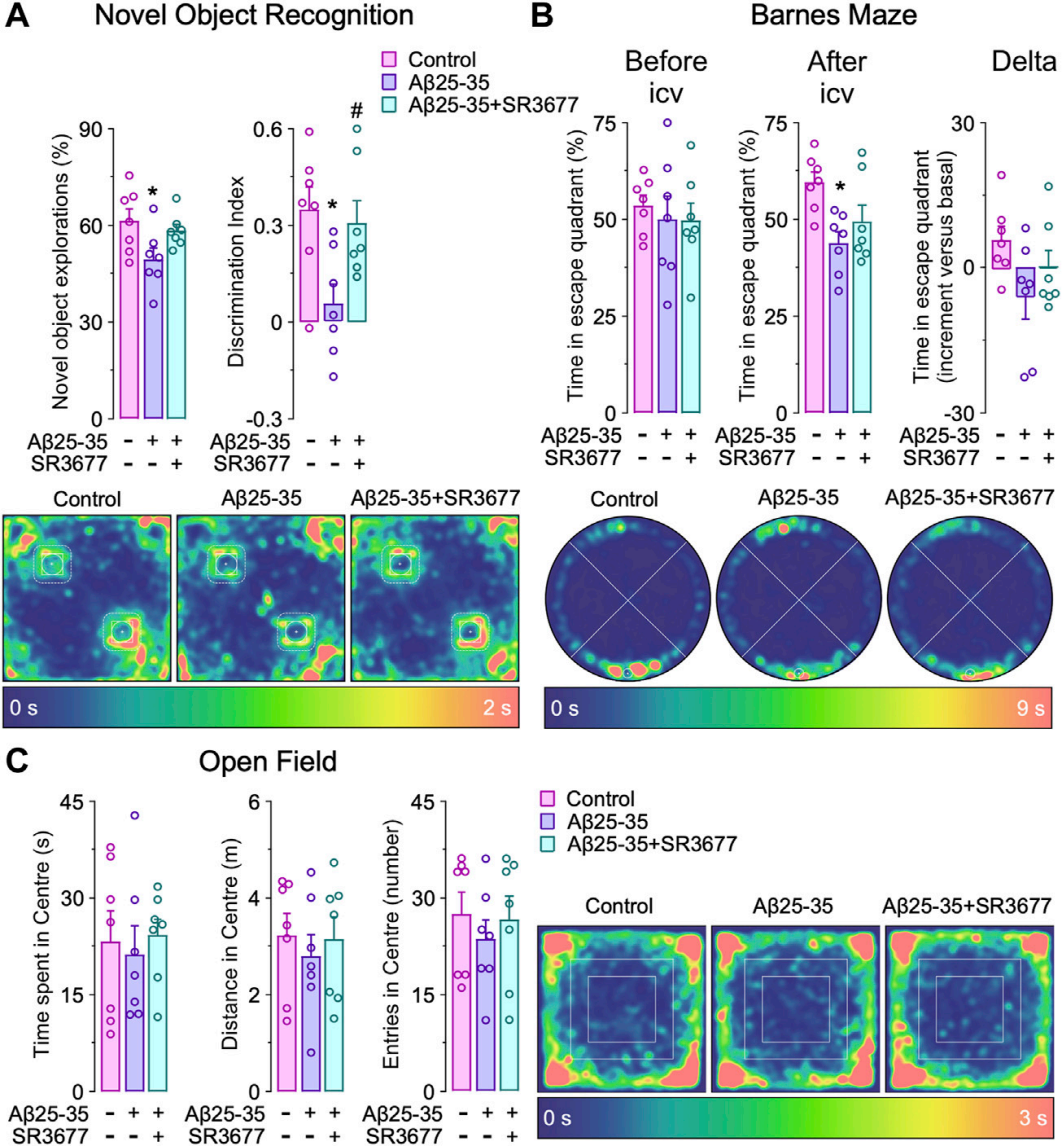
Rock2 inhibition rescues amyloid-β (Aβ)-induced cognitive impairment *in vivo*. Intracerebroventricular stereotactic injections of saline (control) or oligomerized Aβ25-35 (9 nmol) were performed into 12-week-old male mice. When indicated, Rock2 inhibitor SR3677 (2 mg/kg) was intracerebroventricularly injected together with Aβ25-35 oligomers. Behavioral tests were performed at 5 days after injections. **(A)** Novel object recognition test showing novel object explorations and the discrimination index (familiar object in the upper left corner, novel object in the lower right corner). Spatiotemporal quantitative heatmaps are shown. **(B)** Barnes maze test showing time in scape (target) quadrant (lower) before and after Aβ25-35 and SR3677 injections (*n* = 7 male mice per condition). Spatiotemporal quantitative heatmaps are shown. **(C)** Open field test showing the time spent, distance and entries in the center area. Spatiotemporal quantitative heatmaps are shown. Data are mean ± SEM from 7 male mice. *p < 0.05 *versus* control; #*p* < 0.05 *versus* Aβ25-35.

**Rock2 inhibition rescues cognitive impairment induced by Aβ *in vivo*.** Finally, we asked whether the Aβ 25–35-Cdh1-Rock2 pathway herein described takes place *in vivo*. To this end, saline (control) or oligomerized Aβ25-35 were intracerebroventricularly injected in the mouse brain as previously done (Lapresa et al., 2019). One day after injection, we found that both Cdh1 and Rock2 were accumulated in the hippocampus (Figure 5A, left panel); however, at day 3, Cdh1 and Rock2 levels decreased to normal values (Figure 5, middle panel) and, at day 5, Cdh1 levels decreased below controls whereas Rock2 levels were maintained (Figure 5A, right panel). The increase in Rock2 abundance was paralleled by enhanced activity, as revealed by the increase in MBS phosphorylation in the hippocampus that was maintained after 5 days of injection (Figure 5B). To investigate whether Rock2 activation is implicated in the cognitive impairment caused by Aβ25-35, the Rock2 inhibitor SR3677 was intracerebroventricularly administered in the mice together with the Aβ25-35 oligomers. We found that Rock2 inhibition prevented the decrease in the novel object explorations and discrimination index caused by Aβ25-35 (Figure 6A), indicating an improvement in memory performance. In the Barnes maze test, Aβ25-35-injected mice displayed fewer time in scape (target) quadrant than control mice, indicating spatial memory deficits, an effect that was rescued by Rock2 inhibition (Figure 6B). No alterations in the time spent, distance run and number of entries in the center distance were found in the open field test, discarding any motor impairment in this model (Figure 6C). Together, these results indicate that Aβ25-35 enhances Rock2 activity in the mouse hippocampus causing memory loss.

## Discussion

Here, we characterize a novel signaling pathway involved in Aβ neurotoxicity that may open new therapeutic strategies to combat cognitive impairment in AD. We describe that Aβ25-35 oligomers induce cyclin dependent kinase-5 (Cdk5)-mediated phosphorylation of the APC/C-cofactor, Cdh1, leading to inhibition of APC/C and, eventually, neuronal apoptosis. Moreover, we show that Aβ25-35-induced APC/C-Cdh1 inhibition causes Rock2 accumulation and activation in neurons. Finally, we demonstrate that the memory loss caused by Aβ25-35 can be prevented by a pharmacological approach based on the selective inhibition of Rock2 activity. Given that Rock2 accumulates in the neurons of early-stage human AD brain (Herskowitz et al., 2013) and is associated with AD hallmarks (Gao et al., 2019; Cai et al., 2021), our data showing efficacy of the Rock2 inhibitor, compound SR3677, against Aβ25-35 memory impairment in mice should be considered as a potential therapeutic approach in AD.

We herein also characterize the signaling pathway that connects Aβ25-35 with Rock2. It was known that dysregulation of Ca^2+^ homeostasis is a key event in AD pathogenesis (LaFerla 2002) and that Aβ causes p35 cleavage to p25 -a potent Cdk5 activator- in a Ca^2+^-dependent manner (Fuchsberger et al., 2016; Lapresa et al., 2019). Notably, p25 accumulates, and Cdk5 is active, in the brain of AD patients before the onset of clinical symptoms (Patrick et al., 1999). In good agreement with these previous findings, here we demonstrate that the activation of Cdk5 by Aβ is a rapid process, as is the phosphorylation of Cdk5 substrate, Cdh1. Once phosphorylated, Cdh1 becomes disassembled from the APC/C complex, inhibiting its ubiquitin ligase activity for a long period of time. Given that Rock2 is a substrate of APC/C-Cdh1 complex, its inhibition by Cdh1 phosphorylation causes stabilization of Rock2 protein, which is maintained elevated and active also for long periods. Interestingly, this sequence of events mirrors that of Rock2 in AD patients, in which Rock2 is found elevated from the early stages of the disease and remains high throughout the AD progression (Herskowitz et al., 2013). This analogy between our data and those found in humans suggest that the early biochemical changes that we describe, namely Cdk5 activation and Cdh1 phosphorylation, might be considered as potential biomarkers of early AD detection and progression.

Our work also describes an intriguing biphasic effect of the temporal changes in Cdh1 protein abundance by Aβ25-35. Thus, Cdh1 is known to be a substrate of APC/C-Cdh1 and, therefore, when APC/C-Cdh1 is active, Cdh1 undergoes autoubiquitination and proteasomal degradation (Veas-Perez de Tudela et al., 2015b), which is considered a major regulatory system of Cdh1 homeostasis in neurons. Accordingly, in our hands, Aβ25-35 initially triggered APC/C-Cdh1 inactivation, which explains Cdh1 accumulation at the early time points after Aβ exposure, both *in vitro* and *in vivo*. However, at longer time periods, Cdh1 proteins levels underwent a progressive decrease, a result that is compatible with a previous study (Fuchsberger et al.) in which it was found that long-term incubation of neurons with Aβ oligomers causes a proteasome-dependent degradation of Cdh1. These observations suggest that Cdh1 may be subjected to regulation by alternative ubiquitin ligase(s) that could explain the maintained low protein levels of Cdh1. Notably, the F-box and WD-40 domain protein 11, FBXW11, has been observed in the hippocampus of AD mouse models (Sun et al., 2021). Whether FBXW11, which ubiquitinates phosphorylated substrates, including Cdh1 -in proliferative cells (Fukushima et al., 2013)- is responsible for the long-term neuronal degradation of Cdh1, is an interesting possibility that remains to be investigated in the context of AD pathogenesis.

Finally, our data may also provide clues for future development of pharmacological strategies aimed to specifically interfere in the Cdk5-Cdh1-Rock2 axis. Thus, Cdh1 needs to be phosphorylated by Cdk5 to be inactive and, therefore, to maintain Rock2 elevated upon Aβ treatment. These specific Cdk5-dependent phosphorylation sites on Cdh1 are Ser-40, Thr-121 and Ser-163 (Maestre et al., 2008). In fact, Aβ-induced neuronal apoptosis was prevented by expressing a triple phosphodefective (Ser^40^Ala, Thr^121^Ala and Ser^163^Ala) Cdh1 mutant, but not by expressing a triple phosphomimetic (Ser^40^Asp, Thr^121^Asp and Ser^163^Asp) Cdh1 mutant. We propose that these findings may be useful for the design of small molecules aimed to competitively and selectively antagonize these Cdh1 phosphorylation sites. If so, the subsequent disruption of the Cdk5-Cdh1 pathway might eventually serve to prevent aberrant Rock2 accumulation and neurodegeneration in AD.

In summary, our results provide evidence for a key role of the Cdh1-Rock2 signaling pathway in mediating neuronal apoptosis and memory impairment caused by Aβ25-35 oligomers. Thus, Aβ25-35 phosphorylates and inactivates Cdh1, which results in Rock2 stabilization and activation, leading to neurodegeneration. As Rock2 accumulates in the earliest stages of AD and remains elevated throughout the disease progression (Herskowitz et al., 2013), our data set the basis for a future development of therapeutic strategies to overcome neuronal loss and memory impairment in AD.

## Data Availability

The original contributions presented in the study are included in the article/Supplementary Material, further inquiries can be directed to the corresponding author.
