# Supplementary material for: Amyloid-β Induces Cdh1-Mediated Rock2 Stabilization Causing Neurodegeneration
Source: Front Pharmacol. 2022 Apr 14;13:884470. doi: 10.3389/fphar.2022.884470 (PMC9047900; doi:10.3389/fphar.2022.884470)
Supplement: Supplementary file 2 [file DataSheet1.PDF]

Supplementary Material

Amyloid-β induces Cdh1-mediated Rock2 stabilization causing neurodegeneration

Rebeca Lapresa[1,2†], Jesus Agulla[1,2†], Sonia Gonzalez-Guerrero[1,2], Juan P Bolaños[1,2], and Angeles Almeida[1,2*]

[1]Institute of Functional Biology and Genomics, CSIC, University of Salamanca, 37007 Salamanca, Spain

[2]Institute of Biomedical Research of Salamanca, University Hospital of Salamanca, CSIC, University of Salamanca, 37007 Salamanca, Spain

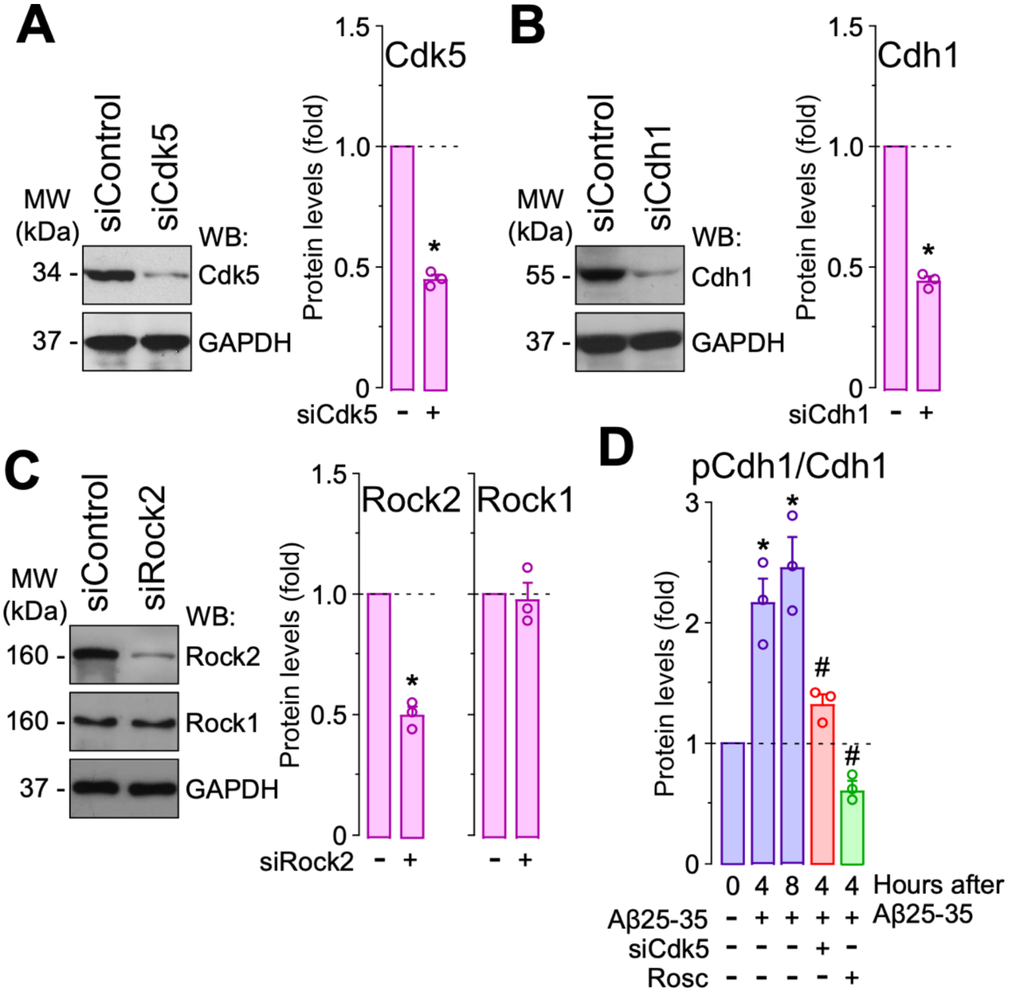

Supplementary Figure 1. The efficacy of small interference RNA (siRNAs) in targeting Cdk5, Cdh1 and Rock2 in neurons. Primary cortical neurons on day 6 in vitro were transfected with siRNA control (9 nM) or with siRNA against **(A)** Cdk5 (siCdk5; 9 nM), **(B)** Cdh1 (siCdh1; 9 nM), or **(C)** Rock2 (siRock2; 9 nM) for 2 days. **(A)** Cdk5, **(B)** Cdh1 and **(C)** Rock2 and Rock1 western blot analysis in neurons at 2 days after transfections (GAPDH, loading control). Western blot bands were quantified by densitometry and data were expressed as the fold change relative to siControl (n=3 neuronal cultures). **(D)** Quatification of western blot bands shown in Figure 1D (n=3 neuronal cultures). Data are mean ± SEM for the indicated number of neuronal cultures. *p<0.05 *versus* (A-C) siControl (D) or 0 hours (D); #p<0.05 *versus* Aβ25-35 (4 hours).

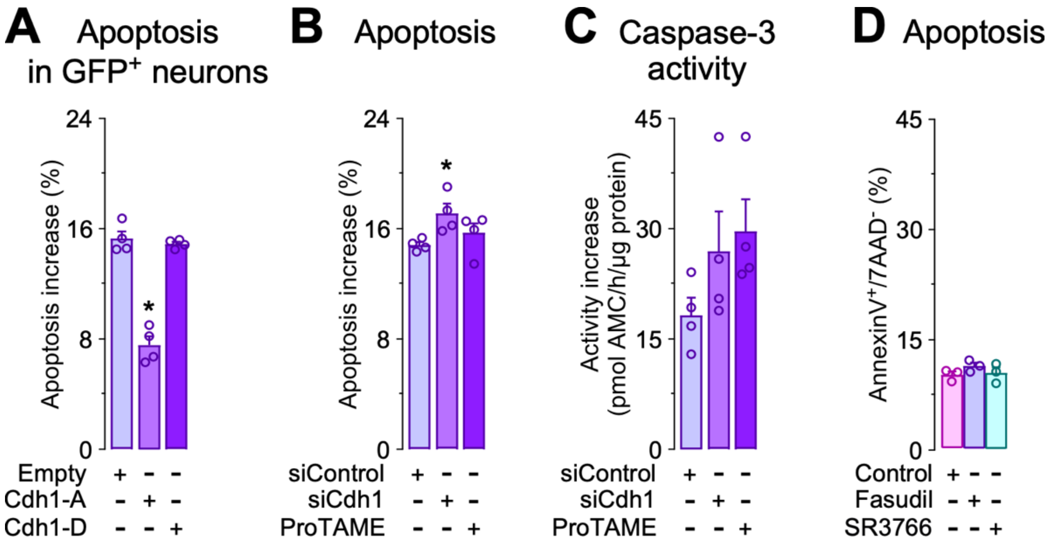

Supplementary Figure 2. Impact of Cdh1 knockdown and APC/C-Cdh1 inactivation in amyloid-β (Aβ)-induced apoptosis. Primary cortical neurons were incubated in culture medium in the absence (control) or the presence of oligomerized Aβ25-35 (10 μM) for 24 hours. When indicated, medium was supplemented with ProTAME (10 μM). **(A)** Neurons on day 6 in vitro were transfected with empty vector or vectors co-expressing GFP and either the phosphodefective (Cdh1-A) or the phosphomimetic (Cdh1-D) forms of Cdh1 and were subjected to Aβ25-35 exposure. Apoptosis was measured by flow cytometry in GFP^+ (transfected) neurons (n=4 neuronal cultures). Data are expressed as the difference in neuronal apoptosis between untreated and Aβ25-35 treated neurons. **(B, C)** Neurons on day 6 in vitro were transfected with siRNA control (siControl; 9 nM) or with siRNA against Cdh1 (siCdh1; 9 nM) for 2 days and then treated with Aβ25-35 oligomerized and proTAME. Data are expressed as the difference in **(B)** apoptosis and **(C)** caspase-3 activity between untreated and Aβ25-35 treated neurons (n=3 neuronal cultures)**. (D)** Neurons were treated with either fasudil (10 μM) or Rock2 inhibitor SR3677 (10 μM). Neuronal apoptosis was analyzed in neurons at 24 hours of treatments (n=3 neuronal cultures). Data are mean ± SEM for the indicated number of neuronal cultures. *p<0.05 *versus* siControl.
